# Corrected analysis of ‘Using financial incentives to promote physical activity in American Indian adolescents: A randomized controlled trial’ confirms conclusions

**DOI:** 10.1371/journal.pone.0233273

**Published:** 2020-06-03

**Authors:** Lilian Golzarri-Arroyo, Xiwei Chen, Stephanie L. Dickinson, Kevin R. Short, David M. Thompson, David B. Allison

**Affiliations:** 1 Department of Epidemiology and Biostatistics, School of Public Health, Indiana University—Bloomington, Indiana, United States of America; 2 Department of Pediatrics, University of Oklahoma Health Sciences Center, Oklahoma City, Oklahoma, United States of America; 3 Department of Biostatistics and Epidemiology, University of Oklahoma Health Sciences Center, Oklahoma City, Oklahoma, United States of America; University of Tennessee Health Science Center, UNITED STATES

In a recent article, Short et al.[1] presented results from an interesting experiment on how financial incentives can potentially increase physical activity among American Indian adolescents, particularly those from the Choctaw Nation of Oklahoma. Adolescents were to be randomly assigned to one of two treatment groups; however, in the case of siblings, the authors made an exception to the randomization by assigning all siblings within a sibship to the same group. While we understand the desire to minimize potential contamination between siblings, this is a violation of the planned individual randomization. Rather, randomization occurred at the level of the family, and it should not be assumed that the siblings are independent subjects. Therefore, it is incorrect to analyze them as independent observations in a regression model [2] as Short et al initially did. Options are either to have only included one sibling from each family, or to account for this correlation in analysis, such as one does in cluster-randomized trials [3,4].

This issue of correlated data is common in many randomized experiments, including students within schools [5] and animals within litters [6–8]. A simplistic way to analyze such cases could be to either only include one observation from each cluster or use the mean of the cluster as the value for analysis, but a better is to include a random effect for the cluster. This way, one can retain all the information and account for the variability within and among clusters.

For this paper, Ms. Golzarri-Arroyo and Dr. Allison contacted the original authors who graciously supplied the raw data including identifiers for siblings (13 pairs) and did so in a timely fashion. We are also grateful for their prompt responses to our questions and for engaging in dialogue about their project.

To first verify the reproducibility of their results, we replicated the published analyses using t-tests and were able to obtain the original results with negligible discrepancies in Means and SD. We also noticed a typographical error in the second to last footnote of Table 1, which should read “Increase from baseline to end of Phase 1 within group, p<0.05”. The error was noted and a correction has since been issued.

**Table 1:** P-values for difference between groups.

	Phase 1		Phase 2	
Outcomes	Short et al.	Golzarri-Arroyo et al.	Short et al.	Golzarri-Arroyo et al.
	t-test	Linear Mixed Model, random effect for family	t-test	Linear Mixed Model, random effect for family
BMI, kg/m2	0.431	0.336	0.179	0.269
BMI, percentile	0.492	0.431	0.447	0.681
Body fat, %	0.823	0.780	0.990	0.857
Fat-free mass, kg	0.448	0.448	0.198	0.216
Waist circumference, cm	0.478	0.428	0.309	0.433
VO2peak, ml/kg FFM/min	0.210	0.251	0.967	0.841
Steps per day	0.848	0.941	0.262	0.354
Glucose, mmol/l	0.726	0.749	0.510	0.335
Insulin, pmol/l	0.965	0.796	0.436	0.593
iHOMA2 (%S)	0.228	0.297	0.845	0.986
HbA1c (%)	0.681	0.794	0.968	0.918
Exercise sessions performed	0.568	0.494	0.469	0.355
Total MVPA time, h	0.942	0.873	0.096	0.102
MVPA time per exercise session, minutes	0.131	0.151	0.002*	0.012*
MVPA time in moderate intensity range, %	0.680	0.716	0.817	0.653
Payments for exercise, USD$	<0.001*	<0.001*	0.043*	0.044*

* p < 0.05

We then performed corrected analyses by performing separate linear mixed models on each outcome. The mixed models included a random intercept for each family in order to account for clustering of outcomes among the 13 sib-pairs. In phase 1 (first 16 weeks) there were 6 sibling pairs in the standard payment group and 7 sibling pairs in the incentive payment group. In phase 2 (second 16 weeks) there were 4 and 5 sibling pairs in the standard and incentive payment groups, respectively. The number of participants was lower in phase 2 than phase 1 due to withdrawal from the study.

Fortunately, our results support the original conclusions and future readers can be assured by these results. The p-values from the results of both analyses are shown in Table 1, where the conclusions of significant results remain the same. There was a significant difference between groups on payments for exercise in both the original and revised analyses, where the treatment group received more payments than the control group in phase 1 (p<0.001 both analyses) and phase 2 (p = 0.043 originally, p = 0.044 revised). Also, in Phase 2, the treatment group had longer MVPA time per exercise sessions compared to the control group (p = 0.002 originally, p = 0.012 revised).

## Supporting information

S1 Data (PDF)

## References

[1] Short KR, Chadwick JQ, Cannady TK, Branam DE, Wharton DF, Tullier MA, et al Using financial incentives to promote physical activity in American Indian adolescents: A randomized controlled trial. PLoS ONE. 2018; 13(6):e0198390 10.1371/journal.pone.0198390 29856832 PMC5983431

[2] Cornfield J. (1978) Randomization by group: A Formal Analysis. American Journal of Epidemiology, 1978; 108(2):100–102. 10.1093/oxfordjournals.aje.a112592 707470

[3] Chuang JH, Hripcsak G, Heitjan DF.(2002) Design and Analysis of Controlled Trials in Naturally Clustered Environments. J Am Med Inform Assoc. 2002; 9(3): 230–238. 10.1197/jamia.M0997 11971884 PMC344583

[4] Bland JM. Cluster randomised trials in the medical literature: two bibliometric surveys. (2004) BMC Med Res Methodol 2004; 10.1186/1471-2288-4-21 15310402 PMC515302

[5] Heo M., Nair S. R., Wylie-Rosett J., Faith M. S., Pietrobelli A., Glassman N. R., et al (2018). Trial Characteristics and Appropriateness of Statistical Methods Applied for Design and Analysis of Randomized School-Based Studies Addressing Weight-Related Issues: A Literature Review. J Obes. 2018; 2018: 8767315 10.1155/2018/8767315 30046468 PMC6036807

[6] Wainwright PE.(1998) Issues of Design and Analysis Relating to the Use of Multiparous Species in Developmental Nutritional Studies. The Journal of Nutrition. 1998; 128:3 Pages 661–663, 10.1093/jn/128.3.661 9482778

[7] Festing MFW, Altman DG. Guidelines for the Design and Statistical Analysis of Experiments Using Laboratory Animals. ILAR Journal. 2002; 43:4 10.1093/ilar.43.1.4 12391400

[8] Abbey H, Howard E. Statistical Procedure in Developmental Studies on Species with Multiple Offspring. Developmental Psychobiology. 1973; 6(4): 329–3 35. 10.1002/dev.420060406 4793362

