# Supplementary material for: Corrected analysis of ‘Using financial incentives to promote physical activity in American Indian adolescents: A randomized controlled trial’ confirms conclusions
Source: PLoS One. 2020 Jun 3;15(6):e0233273. doi: 10.1371/journal.pone.0233273 (PMC7269611; doi:10.1371/journal.pone.0233273)
Supplement: S1 Data — (PDF) [file pone.0233273.s001.pdf]

Choctaw Nation of Oklahoma
Institutional Review Board
OHRP FWA Number: 00001128
OHRP IRB Number: 00004293
One Choctaw Way • Talihina, OK 74571
(918) 567-7000, Ext. 6014

Gary Batton
Chief

Jack Austin, Jr.
Assistant Chief

01/23/2020

Kevin Short, PhD 1200 Children's Ave, Suite 4500
Oklahoma City, OK 73104

RE: Incentivizing Behavior: Promoting More Physical Activity in American Indian Youth
CNO IRB Protocol Number: 12-0162
Editorial Review Approval Date: 01/22/2020

Dear Kevin Short, PhD:

The Choctaw Nation of Oklahoma Institutional Review Board (CNO IRB) reviewed your Other entitled Commentary Choctaw Nation 2019-12-05 at the CNO IRB meeting on 01/22/2020 and was approved.

If you have questions concerning these procedures or need any additional assistance from the CNO IRB, please contact me, Carey Fuller, CNO IRB Administrative Director at 918-567-7000 ext. 6014, or cmfuller@cnhsa.com or David Wharton, CNO IRB Scientific Co-Chair at 580-236-0356 or dfwharton@cnhsa.com.

Sincerely,

John Jones

John Jones, CNO IRB Co-Chair
Choctaw Nation of Oklahoma Institutional Review Board
